# Virtual Reality Simulation in Pediatric Resuscitation for Pre-hospital Providers

**DOI:** 10.7759/cureus.56090

**Published:** 2024-03-13

**Authors:** Emine M Tunc, Derya Caglar, S. Heath Ackley, Rachel Umoren

**Affiliations:** 1 Department of Pediatrics, Division of Emergency Medicine, University of Texas Southwestern Medical Center, Dallas, USA; 2 Department of Pediatrics, University of Washington School of Medicine/Seattle Children's Hospital, Seattle, USA; 3 Department of Neonatology, University of Washington/Seattle Children's Hospital, Seattle, USA

**Keywords:** on-the-job training, paramedic training, pediatric resuscitation, emergency medical service, virtual reality simulation

## Abstract

This technical report explored the feasibility and utility of virtual reality (VR) pediatric resuscitation simulations for pre-hospital providers during their scheduled shifts. To our knowledge, neither the pediatric resuscitation VR simulation nor the feasibility of in situ, on-shift training with VR had been previously evaluated in pre-hospital providers. VR headset was available at an urban city fire station for 10 days where a total of 60 pre-hospital providers were scheduled to work. Providers were made aware of the VR module but no formal demonstration was done. There were no facilitators. Participants filled out an anonymous retrospective pre- and post-survey using a five-point Likert scale, rating their confidence from "not confident" to "very confident" in recognizing and managing pediatric emergencies. We found that VR simulation for pediatric resuscitation was a feasible training tool to use in situ as 63% of the providers were able to use it on shift. Furthermore, self-reported confidence increased after the training where responses of "very confident" increased from 20% to 30% for emergency medical technicians and 55% to 63% for paramedics.

## Introduction

An estimated 1.9 million pediatric transports occur in the US each year [1]. The role of the pre-hospital provider is to provide emergency medical care, stabilize, triage, and transport critically ill patients to an appropriate healthcare setting [2]. When they are called to provide care to an unstable pediatric patient, providers must stabilize and transport the patient to the nearest facility equipped to provide care to pediatric patients. In some cases, these facilities may be a significant distance away [3]. 

Pre-hospital providers may have less comfort with caring for pediatric patients on transport due to limited exposure [4]. Fewer pediatric patients are transported from the pre-hospital setting than adult patients reducing hands-on experience in pediatric care [5]. In addition, the pediatric training received by pre-hospital providers is often far less than the training for the care of adult patients [6]. 

Simulation provides a flexible environment for education through scheduled deliberate practice, the application of knowledge in a controlled environment, and a focus on patient safety [7]. However, the challenges include cost, scheduling, and the need for facilitation [8]. Despite the benefits of simulation training and the desire from pre-hospital providers to implement more of it, previous needs assessment highlighted issues regarding scheduling extra training during off-duty hours [9].

Virtual reality (VR) simulation in healthcare supplements live simulation for both technical and non-technical skills (teamwork, communication, etc.), allowing for the accommodation of different schedules and repeat review of material without the need for a facilitator [10]. Several VR simulation training strategies have been used by pre-hospital providers [11-15] and shown to be effective and comparable to high-fidelity simulation [11]. However, the feasibility of a VR pediatric resuscitation simulation for pre-hospital providers during a work shift and impact on their self-reported confidence in caring for pediatric patients have not been previously evaluated. This technical report seeks to investigate these aspects.

## Technical report

Participants

The participants were pre-hospital providers including emergency medical technicians (EMTs) and paramedics practicing in an urban city fire station in the Pacific Northwest. Participants were identified by their scheduled work shifts and recruited to participate in the study.

Setting and equipment

VR pediatric resuscitation simulations (HealthScholars, USA) were run on the Meta Quest 2 (Meta Inc., USA). The VR module and Meta Quest 2 headset were provided for the duration of the study period by HealthScholars. The module for EMTs, Pediatric Emergency Assessment, included five scenarios: recognition of pediatric stable patient, respiratory distress, shock, supraventricular tachycardia (SVT), and sinus tachycardia. The module for paramedics, Pediatric Emergency Care, included six scenarios: management of pediatric shock, SVT, unstable tachycardia, pulseless ventricular tachycardia, lower airway obstruction, and opioid intoxication. The learner assumed the team lead role in the scenario. Non-player characters (NPCs) of the other team members including an EMT/paramedic partner, respiratory therapist, and compression provider were available to interact by voice command. The team lead could not interact with parents in this program. The NPCs performed tasks specific to their role in the scenario. The program produced a performance score and provided a debrief based on the critical steps that were missed. The performance score was only visible to the participant and was not visible to the study team.

Participants were informed of the availability of the VR simulations by the fire station education officer. The VR headset and instruction manual were available at the fire station for 10 days. The EMTs and paramedics were encouraged to use it once at the beginning of their shift. No formal demonstration was done on its use. For each 24-hour work shift, four EMTs and two paramedics were scheduled. During the study period, 40 EMTs and 20 paramedics were scheduled to work. No facilitator was present. The simulations were available to all the providers during their work shifts without individually assigned scheduled times. Feasibility was defined as the on-shift use of the VR simulations by a majority of the providers who were scheduled to work during the study period. 

An anonymous, retrospective pre- and post-survey was developed by the study investigators. The survey used a five-point Likert scale rating level of confidence in recognition and management of unstable pediatric patients from very confident to not confident. The study was approved as exempt by the Institutional Review Board.

Assessment

Over a 10-day period from October 7 to 17, 2021, 25 (62%) EMTs and 13 (65%) paramedics scheduled to work during the study period were able to complete the scenarios with an overall rate of 63%. All the EMTs and 12 out of 13 paramedics had experienced less than five pediatric resuscitations over the preceding six months. Each participant completed all the scenarios in the module for a total of 125 scenarios for EMTs and 72 scenarios for paramedics.

**Figure 1:**
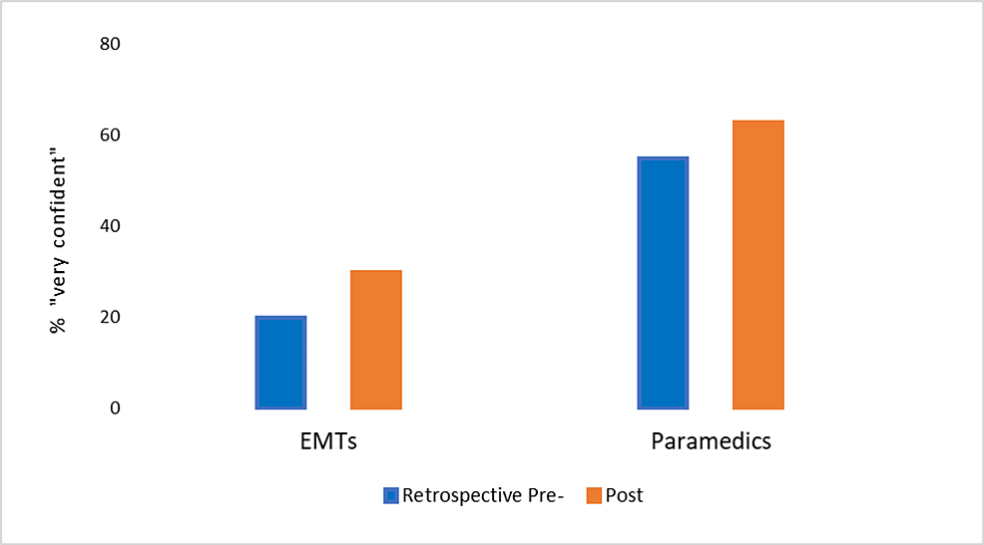
EMT and paramedics’ confidence in recognizing/managing pediatric scenarios after virtual reality simulation. EMT, emergency medical technician.

**Figure 2:**
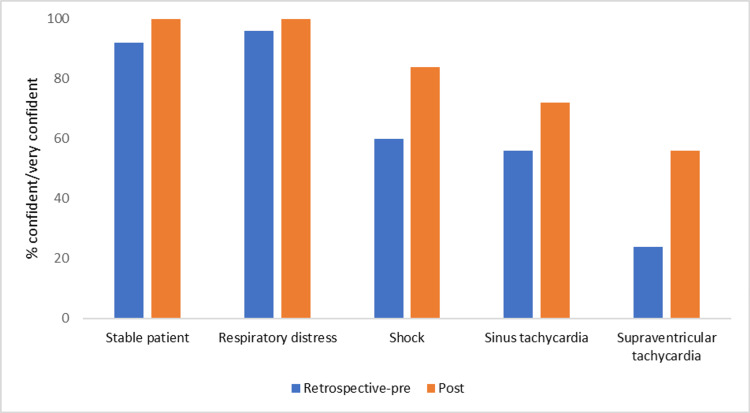
EMTs' confidence in recognizing pediatric scenarios after virtual reality simulation EMTs, emergency medical technicians.

For baseline assessment via a retrospective pre-test, all paramedics rated themselves “confident” or “very confident” with recognizing and managing the pediatric conditions in the scenarios. We analyzed the percentage increase in the response of “very confident” for both groups from the retrospective pre-test to post-test. Responses of “very confident” were higher in both EMTs (increased from 20% to 30%) and paramedics (increased from 55% to 63%) after the training (Figure 1). There were greater increases in EMTs’ confidence (both “very confident” and “confident) with recognition of pediatric shock and SVT compared to other scenarios (Figure 2).

Overall participant perceptions were positive with comments such as “This is an amazing training tool”, “I liked this simulation a lot”, “This has great potential”, and “Awesome tool, think we should work with these more”. Most of the feedback centered around improving the voice command prompts to recognize more commands; for example, “It does need to be refined to have a better understanding of common communication”, “Would like to see a much broader and user-friendly voice recognition setup”, “I got hung up on figuring out the right commands which delayed care”.

## Discussion

This is the first study exploring the feasibility and effect of pediatric VR simulations in pre-hospital providers. We found that the VR simulation was a feasible training tool for EMTs and paramedics with reported increases in confidence in caring for pediatric patients. These findings were similar to those of prior studies and illustrate the acceptability of VR simulations for pre-hospital providers [11-15].

Training during working hours was identified as one of the needs during a needs assessment for pre-hospital provider pediatric training [9]. This study is the first to evaluate the feasibility of the VR training tool while on shift with no orientation or dedicated education time. Sixty-three percent of the pre-hospital providers who were scheduled to work during the 10-day study period were able to complete the modules. This demonstrates the role of VR simulation in improving access to simulation training for pre-hospital providers in situ, on shift.

Our assessment showed an increase in subjectively reported confidence in the evaluation of pediatric patients. This aligns with previous studies done with scenarios related to adult patient care [11,15,16]. While self-reported confidence may not directly translate to clinical practice, more studies are needed to assess the impact on patient care given the real-life experience with pediatric patients is scarce.

VR has several benefits in training healthcare professionals. It allows learners to practice at their own pace and receive feedback without the need for a facilitator. Participants’ comments indicated a positive impression of this education modality. This flexibility is particularly important for pre-hospital providers who often have varying schedules, making standard education difficult and inconsistent. VR also allows for cases to be reviewed multiple times as needed or preferred by the provider.

Our study has several limitations. This was a pilot study on VR feasibility with a small sample size in a single fire station in a busy urban city. The findings may not be generalizable to other locations and settings. We collected self-reported confidence ratings which may not correlate with knowledge base or level of competence in patient care. Further studies with formal pre- and post-assessment of knowledge, skills, and teamwork would be beneficial. In addition, the VR program was lacking in tactile feedback, and the lack of physical interaction with the clinical environment may have an impact on the educational outcomes. Although the VR program provided audio interaction with the avatars of the other team members, this was limited due to the artificial nature of the VR simulation and may not mimic real-life events. Although VR simulation has been shown to be comparable to high-fidelity simulation [11], more studies are needed to compare the use of VR simulations while on shift versus during a scheduled education time to assess the effect of interruptions on learning.

## Conclusions

We evaluated the use of a VR simulation program for pediatric resuscitation used by urban city pre-hospital providers for 10 days. VR modules were used by EMTs and paramedics during their scheduled work shifts. There was no formal demonstration of its use, nor facilitation was provided by a facilitator. We demonstrated that VR simulation is a feasible in situ training tool without a need for a facilitator that efficiently addresses the scheduling constraints associated with coordinating trainees and facilitators for fixed time slots. Moreover, VR simulation has the capacity to enhance confidence in the recognition and management of pediatric illnesses by pre-hospital providers who have limited exposure to pediatric emergencies. Further studies are needed to identify the effects on patient outcomes.
